# Porcine ear necrosis: characterization of lesions and associated pathogens

**DOI:** 10.1186/s13567-023-01218-1

**Published:** 2023-09-29

**Authors:** Mateusz Malik, Koen Chiers, Sebastiaan Theuns, Nick Vereecke, Ilias Chantziaras, Siska Croubels, Dominiek Maes

**Affiliations:** 1 Department of Internal Medicine, Reproduction and Population Medicine, Faculty of Veterinary Medicine, Ghent University, Ghent, Belgium; 2 Department of Pathobiology, Pharmacology and Zoological Medicine, Faculty of Veterinary Medicine, Ghent University, Ghent, Belgium; 3 Laboratory of Virology, Faculty of Veterinary Medicine, Ghent University, Ghent, Belgium; 4 PathoSense BV, Lier, Belgium

**Keywords:** Porcine ear necrosis, lesions, ear biting, mycotoxins, ear tag, behavior, weaned pigs

## Abstract

Porcine ear necrosis (PEN) is characterized by ulcerative lesions of the ear auricle. To investigate that problem, three farms with PEN in nursery pigs were included, and the study aim was to characterize PEN and the potential role of pathogens and mycotoxins. Within each farm, one batch of weaned piglets was included and the prevalence and severity of PEN were monitored for 6–7 weeks. Within each batch, 30 PEN-affected/non-affected animals were randomly selected. Blood samples were taken from these animals, to assess the systemic presence of pathogens and mycotoxins, as well as punch biopsies from the ear auricle for histopathological examination. From 10 animals, scrapings and swabs from the lesions were subjected to nanopore metagenomic sequencing and bacteriological cultivation, respectively. In all three farms, lesions appeared within 3–4 weeks post-weaning. The prevalence at the end of the nursery was 33%, 24%, and 46% for farms A, B, and C, respectively. Most affected pigs had mild to moderate lesions. Blood samples revealed low to very low levels of pathogens and mycotoxins. Different bacteria such as *Staphylococcus*, *Streptococcus*, *Fusobacterium*, *Mycoplasma*, and *Clostridium* species were identified by sequencing in the scrapings. The first two pathogens were also most often identified in bacterial cultures. *Mycoplasma hyopharyngis* was only found in PEN-affected pigs. Histopathological changes were primarily observed in the outer layer of the epidermis. The results suggest that PEN lesions develop by damage to the outer part of the skin e.g. by ear suckling or biting, followed by multiplication of opportunistic pathogens.

**Supplementary Information:**

The online version contains supplementary material available at 10.1186/s13567-023-01218-1.

## Introduction

Porcine ear necrosis (PEN) is characterized by ulcerative, bloody, and wet lesions of the ear auricle, localized mostly on the ear tips [1]. Different names have been used for this condition such as ear-tip necrosis (ETN), ear-biting, porcine ear necrosis syndrome (PENS), ulcerative spirochetosis, or streptococcal auricular dermatitis. The condition should be considered a welfare problem, especially in case of severe lesions. A recent study showed that mild PEN lesions did not affect pig growth [2] but likely severe PEN lesions do decrease performance. In addition, the skin wounds at the ears may serve as an entry point for opportunistic bacteria, as has been shown for tail-biting lesions [3]. Such bacteria may subsequently spread throughout the body and cause abscesses in the lung and other internal organs, leading to condemnations of the carcasses at slaughter [4].

The cause and pathophysiology of PEN are largely unknown. Three major pathogenesis hypotheses have been described in scientific literature thus far: (1) obstruction of small blood vessels due to cold agglutinins [5]; (2) damage of the epidermis caused by staphylococcal exfoliative toxins; and (3) ear-biting leading to skin injury followed by β-hemolytic streptococcal infection [6, 7]. Spirochetes of the genus *Treponema* have also been associated with PEN [8]. However, experimental intradermal inoculation of *T. pedis* did not result in PEN lesion formation [9]. Porcine circovirus type 2 (PCV2) infections were mentioned by Pejsak and colleagues [10] as a possible risk factor, though no direct influence was shown so far. Since PCV2 frequently infects nursery pigs and might exert immunosuppressive effects, its significance in herds affected by PEN is questionable. Recently, Costa et al. [11] could reproduce PEN via intradermal inoculation of suspensions made from tissue collected from fresh PEN lesions, but only mild lesions were found.

Several non-infectious risk factors have been suggested including high stocking density, low availability of feeders and drinkers, mycotoxin contamination of feed, fully slatted floors, high humidity, and high environmental temperature [6, 12]. A recent study including different successive batches of nursery pigs [2] reported that pens with a high and low prevalence of affected pigs can exist next to each other, and that lesion prevalence in specific pens is not consistent over time. This suggests that pigs affected with PEN are not always present in specific “high-risk” pens over successive batches.

So far, the ear lesions of pigs affected with PEN have not been fully characterized neither were the pathogens involved assessed using more recent and advanced diagnostic procedures. To further elucidate the pathogenesis of the condition, the aims of the present study were to investigate PEN lesions in nursery pigs of different farms, to characterize the lesions and to assess the pathogens involved using nanopore metagenomic sequencing and bacterial culture.

## Materials and methods

The study protocol was approved by the Ethical Committee of the Faculty of Veterinary Medicine and the Faculty of Bioscience Engineering, Ghent University (EC2020-095), as well as by the Flemish governmental agency for animal welfare (DWZ/KF/21/1.15/40).

### Study design and farm characteristics

Three commercial single-site farrow-to-finish pig farms were included. The farms had been suffering from PEN in nursery pigs for more than 1 year, as confirmed by the farmer and the farm veterinarian. The research was conducted between May and November 2021. On each farm, one entire batch of piglets was observed from weaning to the end of the nursery, spanning a period of 6–7 weeks. The weaning age was 21 days in farm A, and 28 days in farms B and C. The farms were visited by the first author to collect appropriate farm data using a questionnaire and to evaluate the severity of the problem. The general characteristics obtained by the questionnaire are summarized in Additional file 1. The number of nursery pens that were monitored during the study differed between farms. In Farm A, 12 pens (on average 40 pigs/pen) were divided over three compartments, in Farm B, 14 pens (38 pigs/pen) were in one compartment, and in Farm C, 12 pens (25 pigs/pen) were present in the same compartment. The farms were visited weekly, and the prevalence and severity of PEN of the entire weaning group were assessed. During the last two visits, various samples namely scrapings (10/farm), swabs (10/farm), and biopsies of the ears (30/farm), plasma (30/farm), and serum (18–30/farm and pooled by 6, depending on present severity score) were collected for further analysis, including metagenomic sequencing, bacterial culture, histopathology, and blood for mycotoxins analysis. The samples were taken towards the end of the nursery, as at that time, lesions of different severity were present and could be sampled. There were no other animal health problems on the farms during the study.

### Assessment of PEN prevalence and severity

In all three farms, every pen of the weaning group was monitored weekly, and the animals were restrained individually in the pen, and visually evaluated one by one for the presence and severity of PEN lesions. This also allowed us to assess the distribution of the total number of affected animals over the different pens in the different compartments. A five-point scoring system to assess the severity of the lesions was used as described previously [7]. In short, the scores (0 to 4) corresponded to the following conditions: 0 = no deviations, 1 = incipient red discoloration or a crust at the tip of the ear, 2 = more black-like discoloration and a rounded ear tip, 3 = severe necrosis with a part less than one-third of the ear missing at the tip, and 4 = piglets lost more than one-third of the ear. After performing the evaluations on farm A, it was decided to record other lesions (scratches/small wounds) different than PEN on both ears from each individual animal on farms B and C. This was done at all scoring timepoints. Piglets were ear tagged on all farms (official ear tag with farm number and country code) but in farm C, ear tags were consistently placed at the same side of the animal. This allowed us to evaluate the effect of the ear tag on PEN lesions in that farm.

### Sampling

In total, 1280 piglets were evaluated in this study. The number of pigs in the entire weaning batches of the three farms were as follows: farm A 485, farm B 527, and farm C 268 piglets. Within each farm, pigs with lesions of different severity were sampled.

Blood samples were collected via jugular vein puncture from 18 to 30 randomly chosen animals with different lesion severity scores 2 weeks before the end of the nursery period. Clotted blood samples were centrifuged for 20 min at 2000 × *g* and sera were collected. Within each farm, individual samples of six animals were pooled based on the PEN severity score present on the farm. To this end, on farms A and B, three pooled samples were prepared (from pigs with score 0, 1, or 2), and on farm C, five pooled samples were obtained (from pigs with score 0, 0, 1, 2, or 3). Blood of 30 animals per farm were taken to obtain plasma for further mycotoxins analysis.

To investigate possible differences in the microbiota present in the lesions from the animals that were blood sampled in each farm, 10 of them were selected at random to obtain scrapings namely 6 from affected (all severity scores) and 4 from non-affected animals. Scrapings of the lesions and underlying tissue from PEN-affected animals, as well as ear tip skin scrapings of unaffected animals, were collected with a scalpel blade. The lesions or the area around the lesions was not cleaned before sampling. On farms B and C, 5 scraping samples were taken 1 week later. The later sampling likely had only minor or no effect on the results.

### Metagenomic analysis

Serum and scrapings were analyzed using nanopore metagenomic sequencing as described previously [13, 14]. In short, (pooled) serum samples were pre-purified using a patented sampler (EP 19183233.6). Scrapings were crushed in dPBS using a 1.5 mL Eppendorf tube squisher (Zymo H1001) and filtered through a 1.5 mL Eppendorf tube 0.8 μm centrifuge filter (Vivaclear, Sartorius) at 2000 rpm for 5 min. The resulting filtrates were subjected to enzymatic host depletion and *ad random* amplification procedure. The resulting DNA was subjected to rapid library preparation using the RBK096 library prep (ONT), multiplexing up to 24 samples per run. Sequencing was performed on R9.4.1 flow cells (ONT) using the GridION device, which allows real-time data acquisition, super accurate base calling, and demultiplexing via Guppy (v.6.1.5). The reads were taxonomically classified using in-house validated bioinformatics pipelines. A spike-in virus was used to perform normalization between samples and to give a semi-quantitative report as described before [15]. This allowed us to report both viruses and bacteria in five categories, including very low, low, medium, high, and very high. The bacterial classification was limited to the genus level as previously discussed [14]. If two or fewer absolute reads were classified, they were not reported [13, 15].

### Bacteriology culturing

The animals that were selected for scrapings sampling for nanopore metagenomic sequencing were also selected for bacteriological culture. The swabs were taken just after, and from the same area of the ears as was done for the scrapings. They were collected with cotton sterile swabs with Amies transport medium, and transported within 4 h to the Animal Health Care Flanders laboratory (DGZ Vlaanderen, Torhout, Belgium), where standard aerobic and anaerobic agar culturing was performed. Namely for the aerobic pathogens Columbia sheep agar, MacConckey agar, and Columbia horse agar were used; which were incubated for 48 h at 37 °C in CO_2_ (7.5%). For the anaerobic pathogens, fastidious anaerobic agar and Colombia agar were used, which were incubated for 24 h in anaerobic conditions (AnaeroGen, Thermo Scientific, USA). The samples were inoculated on the media until a pure culture was obtained. These pure cultures were identified using MALDI-TOF. The bacterial number or load in the sample was expressed semi-quantitatively namely: low (yellow) 0–10 colonies on plate, intermediate (orange) 11–50 colonies, and high (red) > 50 colonies on plate.

### Histopathology

Six millimeters punch biopsies were collected from the same 30 animals per farm that were blood sampled. From the affected animals, a biopsy was taken from the margin of the lesion. From non-affected animals, a biopsy was taken from the ear tip, 5–10 mm from the ear edge. The collected samples were first fixed in 4% neutral buffered formalin and embedded in paraffin before being stained with hematoxylin and eosin followed by microscopic evaluation.

### Mycotoxin analyses and feed composition

Blood samples from 90 animals were also collected in EDTA tubes (uncoagulated blood). The samples were centrifuged for 10 min at 4 °C at 3725 × *g* to obtain plasma, which was then stored at −20 °C until further analysis. The plasma samples were analyzed using liquid chromatography combined with triple quadrupole tandem mass spectrometry. The analysis has been validated for pig plasma as a multi-mycotoxin LC–MS/MS method [16] to assess the presence of mycotoxins, with phase I and II metabolites simultaneously. The following toxins were included: 3-acetyl deoxynivalenol, 15-acetyl deoxynivalenol, alternariol, alternariol monomethyl ether (AME), aflatoxins: B1, B2, M1, M2, G1, G2, beauvericin, de-epoxy-deoxynivalenol, deoxynivalenol (DON), deoxynivalenol-glucuronide, deoxynivalenol-sulfate, enniatins: A, A1(ENNA1), B (ENNAB), B1 (ENNAB1), fumonisins (FBs): fumonisin B1 (FB1), B2, B3, HT-2 toxin, ochratoxin A (OTA), tenuazonic acid (TEA), T2 toxin, zearalenone (ZEN), α-zearalanol, β-zearalanol, α-zearalenol, β-zearalenol, α-zearalenol-glucuronide, β-zearalenol-glucuronide, zearalanone, zearalenone-glucuronide and zearalenone-sulfate.

Additionally, two feed samples were collected in each farm, one from the feed that was provided during the first week post-weaning, and a second from the feed that was provided during the fourth week post-weaning. Each sample was obtained by pooling feed samples from different (four to six) feeding troughs throughout the compartment. The samples were stored at room temperature, and the presence of DON, FBs, and ZEN was determined by a multi-mycotoxin method—LC–MS/MS.

### Drinking water quality

On each farm, water samples were taken from the drinking nipple in the stable and transported within 24 h to the DGZ Laboratory. After 30 s of continuous flow from the nipples, the water sample was collected into specific bottles provided by the laboratory. Bacteriological (the number of coliforms, intestinal enterococci, sulfite-reducing clostridia, overall aerobic bacteria) and chemical (ammonia, nitrates, sulfates, hardness, and salinity concentration) analyses were performed.

### Stable climate

While carrying out the study on farm A, it was decided to assess as additional descriptive information different stable climate parameters in farms B and C. To this end, loggers were placed in the middle of the compartment, at a height of one meter above the ground. The following parameters were measured every 30 min using a Tinytag Plus2 logger (Gemini Data Loggers, UK): ambient temperature (°C), relative humidity (%), and dew point (°C).

### Statistical analysis

A generalized linear mixed model (logistic regression with a binomial probability distribution) was run to determine the effect of wearing an ear tag and the effect of the week, on having a scratch or not in the first 3 weeks after weaning on farm C. The effects of pen and pig were included in the model as random effects.

Additionally, a cumulative odds ordinal logistic regression with proportional odds (generalized linear mixed model) was run to determine the effect of wearing an ear tag (present on the left or right ear) and the effect of the week, on having a higher PEN score. The effects of pen and pig were included in the model as random effects.

Also, we performed a non-parametric test (Mann–Whitney test) to check whether the presence of PEN lesions was associated with the levels of mycotoxins (as tested in plasma samples).

A cumulative odds ordinal logistic regression with proportional odds was run to determine the effect of a higher number of mycotoxins on having a higher severity score for PEN. The farm was also considered in the model (fixed effect).

Finally, we made use of a non-parametric test (Spearman’s rho) to check the correlations between PEN severity and the load for all viruses or bacteria revealed in the metagenomics analysis.

Statistical analysis was performed using IBM® SPSS® Statistics for Windows Version 29 (IBM Corp., Armonk, N.Y., USA). *P*-values below 0.05 were considered statistically significant.

## Results

### PEN prevalence

**Figure 1:**
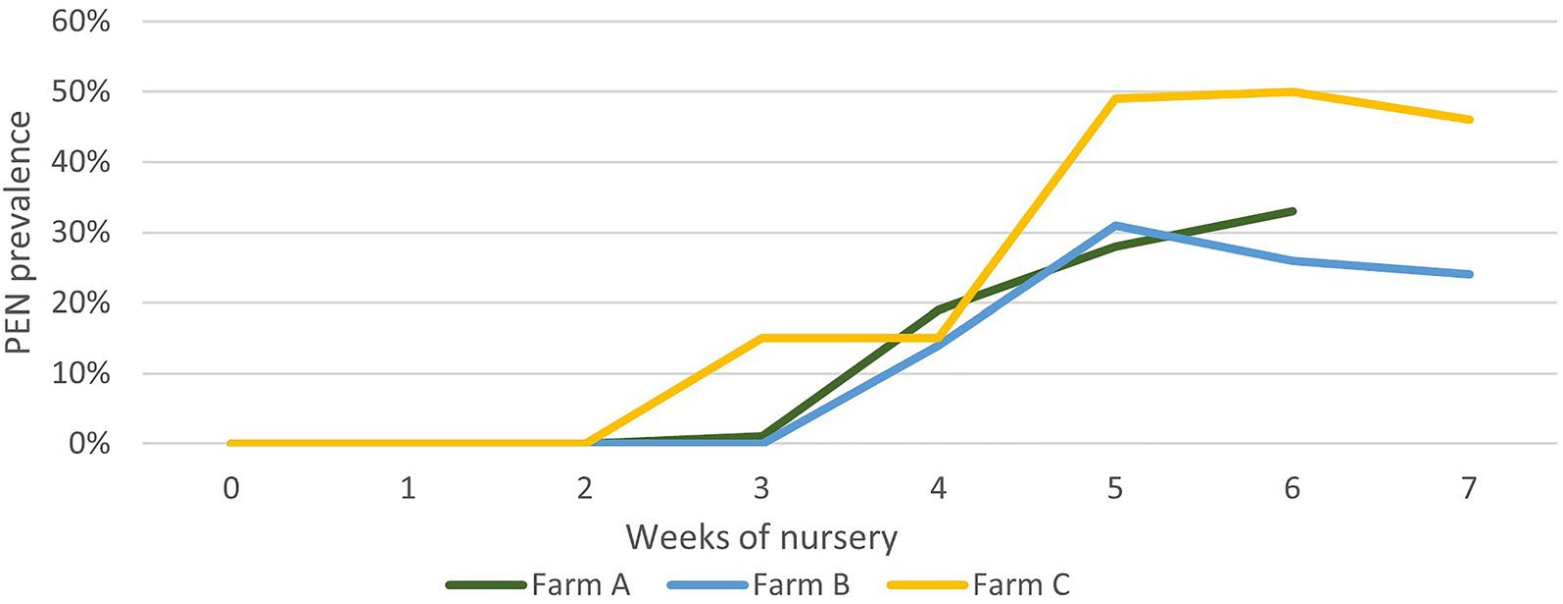
**Weekly prevalence of porcine ear necrosis (PEN) lesions in nursery pigs from three farms**. The number of piglets at weaning on farms** A**,** B**, and** C** was 485, 527, and 268, respectively.

The entire weaning batch in farms A, B, and C included 485, 527, and 268 piglets, respectively. Figure 1 depicts the weekly prevalence of PEN. No animals showed signs of PEN at weaning. The prevalence of PEN in each batch increased with the number of weeks post-weaning. The maximum prevalence was 33%, 31%, and 50% in farms A, B, and C, respectively, what was reached at 5–6 weeks post-weaning. In the final 2 weeks of the nursery, the prevalence in farms B and C decreased slightly. The mortality on farms A, B and C was 2.7%, 0.8%, and 1.5%, respectively.

### PEN prevalence by location in the stable

**Figure 2:**
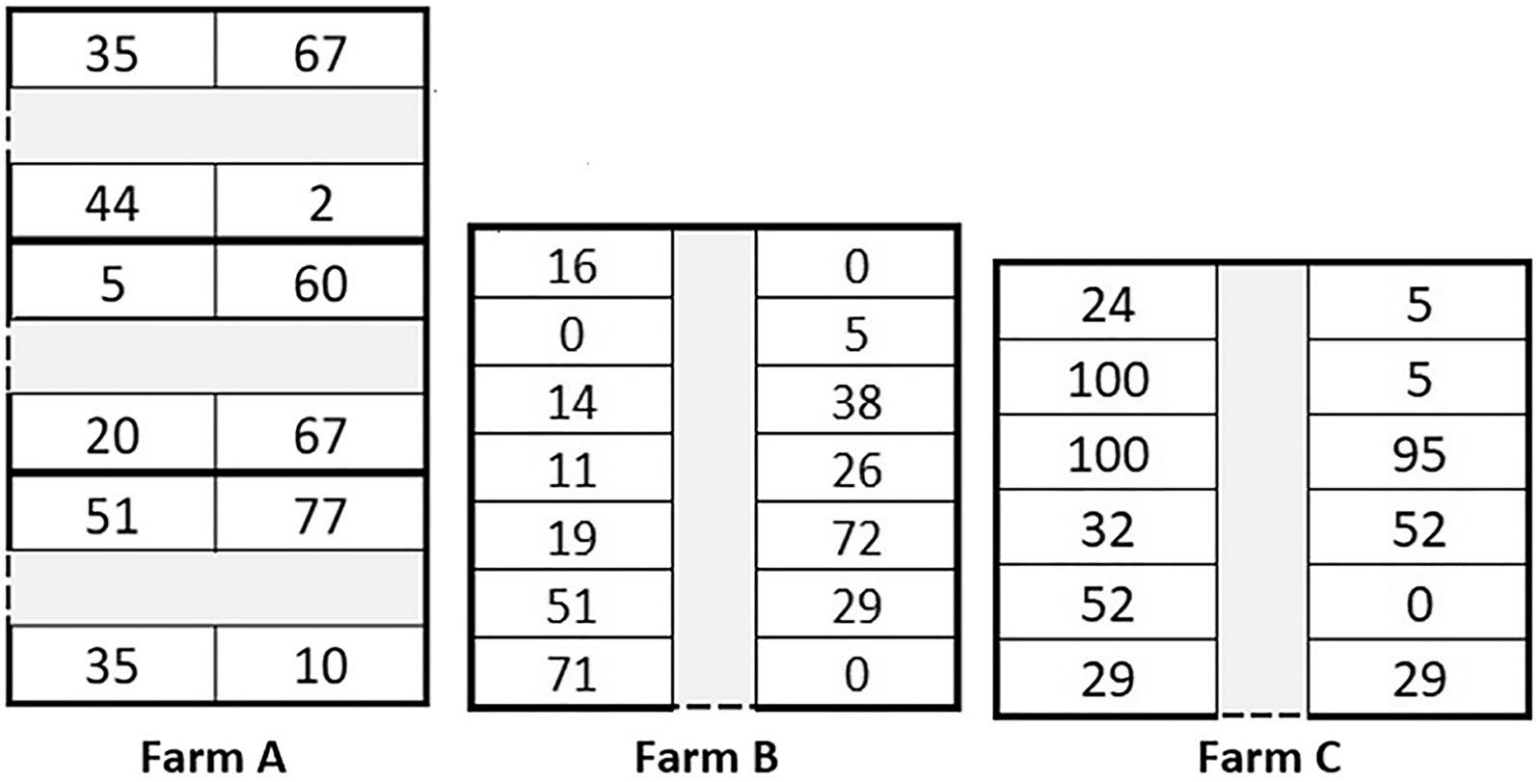
**Percentage of animals affected with porcine ear necrosis per pen, at the end of nursery period in farms A, B, and C**. The mean number of pigs per pen was 40, 38, and 25 in farms** A**,** B**, and** C**, respectively.

Figure 2 shows an outline of the pens in the compartments as well as the percentage of affected animals in each pen at the end of the nursery period. The prevalence of affected animals per pen ranged from 2 to 77%, 0–72%, and from 0 to 100% in farms A, B, and C, respectively.

**Figure 3:**
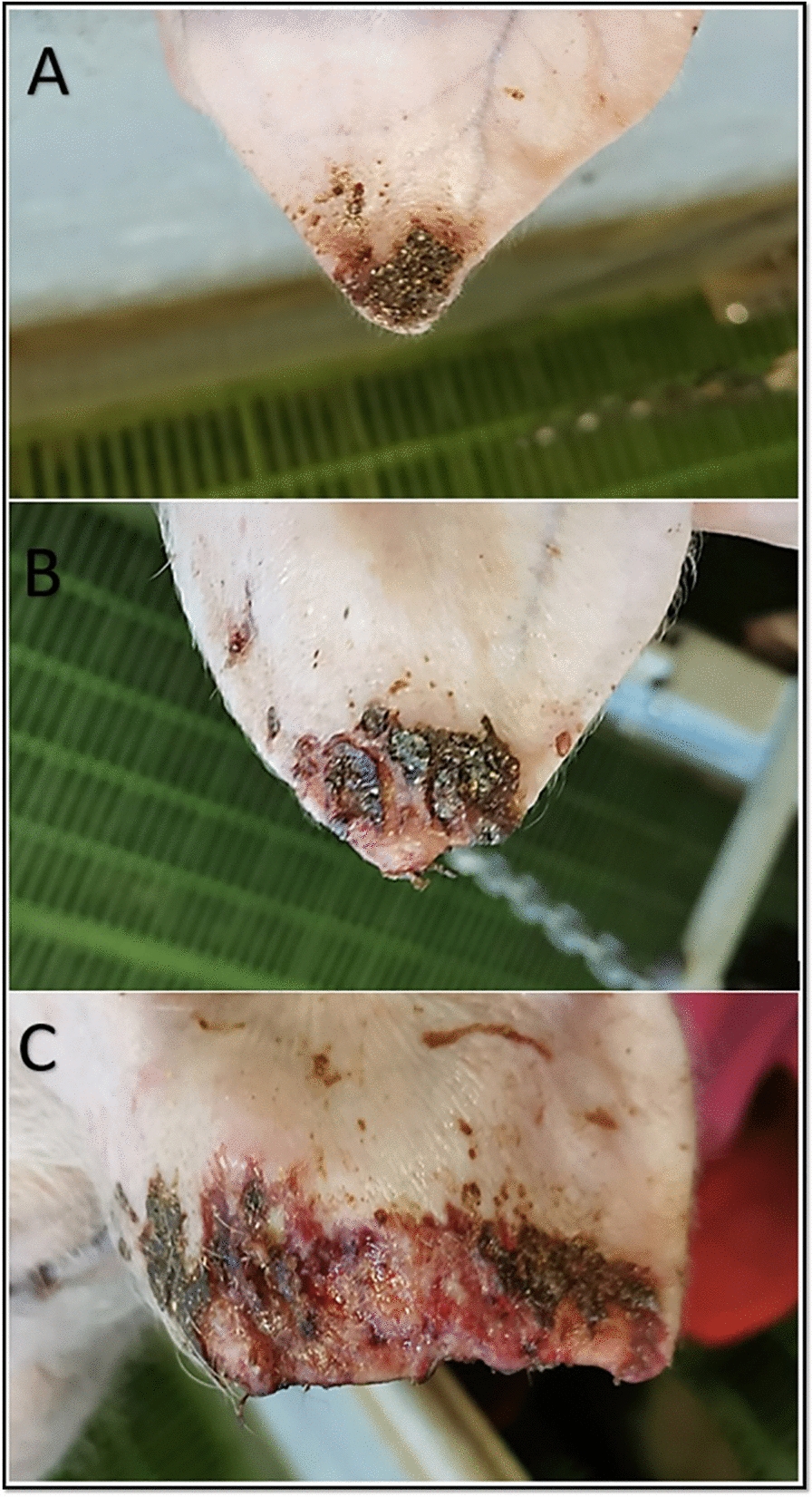
**Example of ear lesions evaluated during the study**.** A** Piglet with mild lesions (score 1)—light reddening and small crusts, **B** ear of a moderately affected piglet (score 2)—rounded ear tip with crusts and wet tissue beneath, **C** ear of severely affected piglet (score 3)—partially missing ear pinna, with remaining crusts and fresh wet tissue.

The lesions in animals from all three farms developed first as a dry small crust on the ear tip or as reddening with edema and a small dry wound at the tip (Figure 3A). Some of these lesions progressed from moderate (Figure 3B) to severe wet wounds with partial ear pinna loss. The tissue beneath was often fresh and moist (Figure 3C).

**Figure 4:**
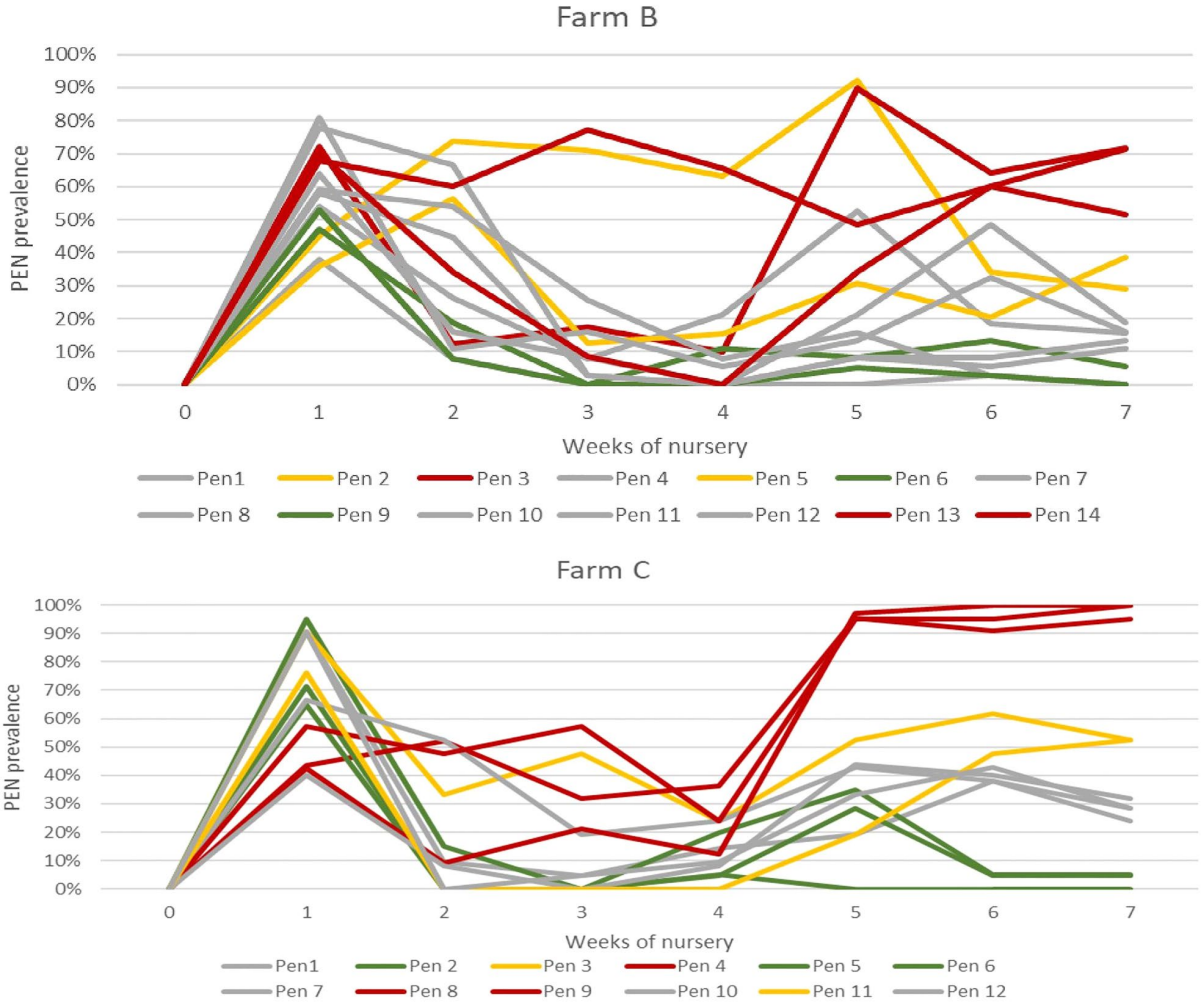
**Weekly prevalence of all recorded ear lesions/alternations recorded at the pen level on farms B and farm C**. Colors of the lines related to the prevalence: red—very high, yellow—high, grey—moderate, green—low.

In the first week post-weaning in farms B and C, small scratches/wounds were present in 80–95% of the pigs (Figures 4A, B). The prevalence decreased to less than 20% in most of the pens (20/26) 2–3 weeks post-weaning. In the six remaining pens where the prevalence of scratches/wounds remained 20% or higher, four pens reached the highest PEN prevalence (95–100%) at the end of the nursery.

In farm C, the ear tags were placed in the right ear only, 2–3 cm from the edge and tip of the ear. The prevalence of all small wounds/scratches after the first week post-weaning (*n* = 269 pigs), was 26% and 62% in the right and left ear, respectively. In pen numbers 3, 4, 7, and 8, the prevalence of wounds/scratches did not decrease below 20% in week 2–3 post-weaning (*n* = 66 pigs), and the average prevalence for the first 3 weeks was 11% (SD ± 8%) and 60% (SD ± 17%) in the right (with the ear tag) and left ear (without ear tag), respectively.

The odds of having a scratch on an ear without an ear tag was 7.42 (95% CI 5.20 to 10.62) times that for ears with an ear tag, t = 10.998, *p* < 0.001. Week 1 was associated with an increase in the odds of having a scratch when compared with Week 3, with an odds ratio of 10.01 (95% CI 6.95 to 14.42), t = 12.384, *p* < 0.001. Week 1 was associated with an increase in the odds of having an increase in scratches when compared with Week 2, with an odds ratio of 10.42 (95% CI 6.95 to 14.42), t = 12.49, *p* < 0.001. Week 2 was not associated (t = −0.206, *p* = 0.837) with a change in the odds of having a scratch when compared with week 3, with an odds ratio of 0.96 (95% CI 0.63 to 1.46).

### Severity of the PEN lesions

The results of the severity scoring of the piglets in the three farms are shown in Table 1. On farms A and B, only 1–3% of the pigs had a lesion score of 2, whereas, on farm C, 34% of animals developed lesions with a score of 2 or 3. Very severe lesions with a score of 4 were not found in any of the farms.

**Table 1:** **Distribution of different lesion scores in nursery pigs of the three farms at the end of the nursery**.

Score	Farm A	%	Farm B	%	Farm C	%
0–4	*n* = 472	100	*n* = 523	100	*n* = 262	100
0	314	66	393	75	140	53
1	154	33	115	22	35	14
2	4	1	15	3	62	24
3	0	0	0	0	25	10
4	0	0	0	0	0	0

In animals with different scores on both ears, the highest score was counted.

In farm C where the ear tags were always placed on the right ear, for the last 3 weeks of the nursery, the average PEN severity score was assessed ear separately for the left and right in three pens with the highest PEN prevalence. The average severity scores were 1.06 (SD ± 0.31) and 1.75 (SD ± 0.20) for the ears on the right and the left side, respectively (*n* = 135). In those pigs, the average PEN prevalence severity score of 3 at the end of the nursery was 0% and 29% on the right and left ears, respectively.

The odds of having a higher PEN score on an ear without an ear tag was 2.42 (95% CI 1.82 to 3.22) times that for ears wearing an ear tag, t = 6.05, *p* < 0.001. Week 7 was associated with an increase in the odds of having an increase in PEN score when compared with Week 5, with an odds ratio of 1.77 (95% CI 1.31 to 2.38), t = 3.764, *p* < 0.001. Week 6 was associated with an increase in the odds of having an increase in PEN score when compared with Week 5, with an odds ratio of 1.75 (95% CI 1.30 to 2.36), t = 3.694, *p* < 0.001. Week 7 was not associated (t=-0.77, *p* = 0.939) with a change in the PEN score when compared with Week 6, with an odds ratio of 0.99 (95% CI 0.74 to 1.32).

### Metagenomic profiling of viruses and bacteria

Overall, many different pathogens were detected in the serum by nanopore metagenomic sequencing, mostly at low and/or very low levels. Porcine Parvovirus (PPV) type 5 (3/3 pooled samples in farm B) and PPV type 2 (4/5 pooled samples in farm C) were found to be present at high levels. Also, torque teno sus virus (TTSuV) was found on all three farms (farm A: 3/3; farm B: 3/3; farm C: 3/5), but at low levels. The porcine reproductive and respiratory syndrome virus (PRRSV), and porcine bocaparvovirus, were present at low levels. For the bacteria, *Streptococcus* sp. was found in one pooled sample from farm B and one from farm C.

For the ear scrapings, the most prevalent viruses were Bocaparvovirus (27/30, ranging from very low to medium levels), porcine parvoviruses, including type 2 (2/30, very low to low), 5 (5/30, very low to high), 6 (1/30 very low), and 7 (1/30 very low), TTSuV (4/30, very low to low), swine pneumovirus (4/30 very low), porcine cytomegalovirus (1/30 very low), PRRSV (1/30 low), porcine polyomavirus (1/30 very low), and porcine adenovirus 5 (1/30 very low). Also, other viruses (kobuvirus, rotavirus, picobirnavirus, enterovirus) were detected in various samples (Additional file 2).

**Figure 5:**
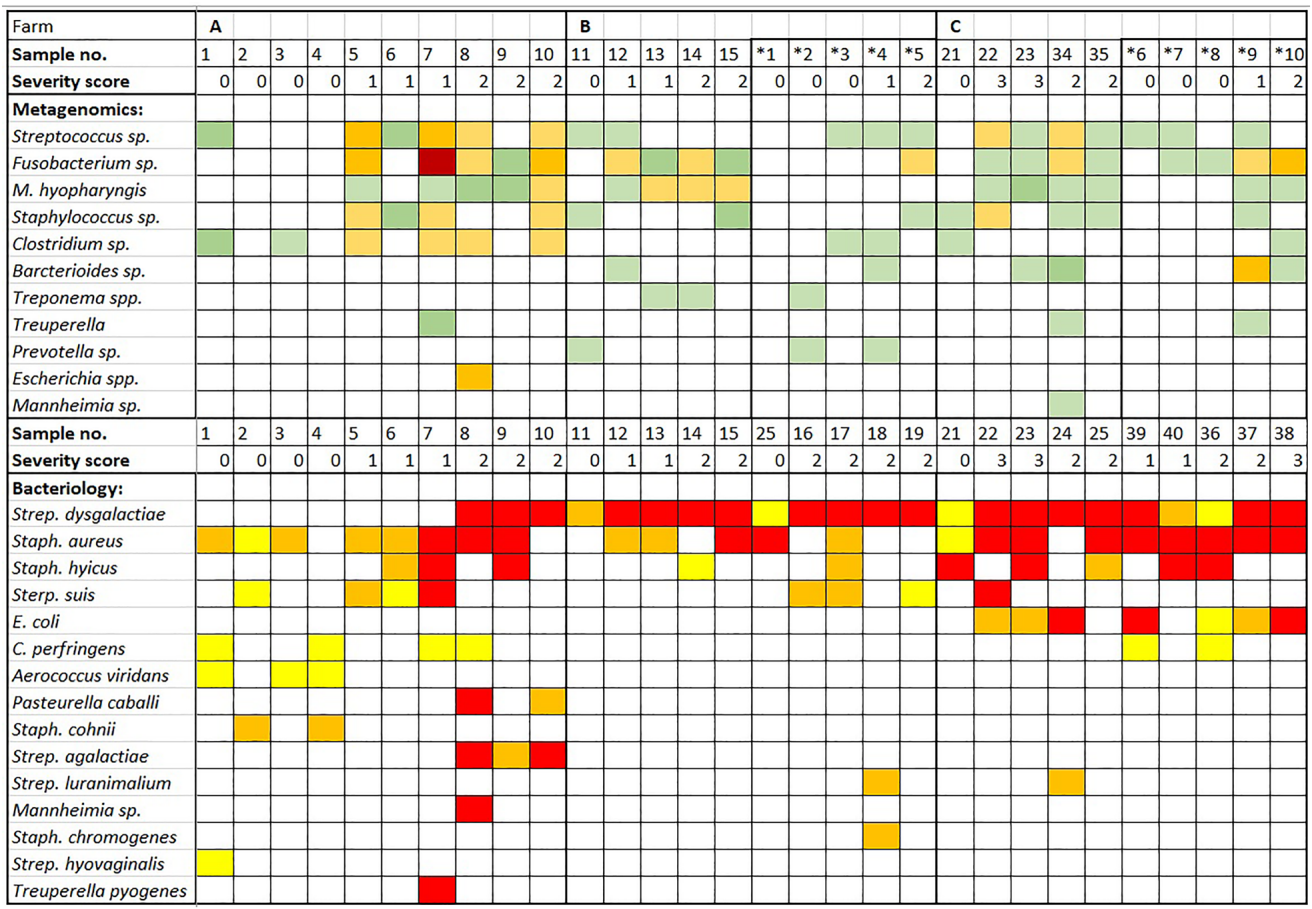
**Heatmap of bacteria found in scraping samples, with their loads, analyzed by nanopore sequencing (white—negative, light green—very low, green—low, light orange—moderate, orange high, red—high loads) together with the bacteriology results load (white—negative, yellow—low, orange—moderate, red—high)**. *Samples with the asterisk were collected 1 week later.

The most prevalent bacterial genera in scrapings were *Fusobacterium* sp. (18/30, very low to very high; likely *F. necrophorum*), *Streptococcus* sp. (17/30, very low to high), *Mycoplasma* sp. (15/30, very low to medium; likely *M. hyopharyngis* when considering full-length 16 S rRNA gene sequences), *Staphylococcu*s sp. (12/30, very low to medium), and *Clostridium* sp. (10/30, very low to medium). Some less abundant bacterial genera were *Campylobacter* sp. (5/30, very low to medium; likely *C. mucosalis* when considering full-length 16S rRNA gene sequences), *Trueperella* sp. (3/30, very low to low), *Porphyromonas* sp. (3/30 very low), *Treponema* sp. (3/30 very low), *Actinobacillus* sp. (3/30, very low to medium), *Mannheimia* sp. (1/30 very low), and *Escherichia* sp. (1/30 medium). Also, other bacteria were identified, including *Prevotella* sp., *Bacteroides* sp., *Neisseria* sp., and some Lactobacillaceae. *Mycoplasma hyopharyngis* was also the only bacterium present in PEN affected pigs (75%) and absent in PEN-negative pigs (100%). The number of different viruses and bacteria detected on farms A, B, and C were 13, 11, and 17, respectively. Figure 5 shows a heatmap of selected bacteria found in the ear-scraping samples of the nursery pigs from the three farms, along with their semiquantitative abundance, and ranked by prevalence. Complete output files (viruses and bacteria) are shown in Additional file 2.

The only pathogens showing a statistically significant correlation to PEN lesions were: *M. hyopharyngis* (0.590, *p* < 0.001), Porcine Parvovirus 2 (0.438, *p* = 0.015), *Fusobacterium* spp. (0.404, *p* = 0.027), Rotavirus C (0.385, *p* = 0.035).

### Bacteriology

Bacteriological culture of skin and lesion swabs revealed the growth of *Staphylococcus aureus* (*S. aureus*) and *hyicus* (*S. hyicus*), *Streptococcus suis* (*S. suis*), and *Streptococcus dysgalactiae* ssp.* equisimilis* (*S. dysgalactiae* ssp.* equisimilis*) as well as *Escherichia coli* (*E. coli*) in both PEN-affected and non-affected pigs. *Clostridium perfringens*, *Streptococcus hyovaginalis*, *Streptococcus agalactiae*, *Staphylococcus cohni*, *Staphylococcus sciuri*, *Aerococcus viridans*, *Pasteurella caballi*, *Trueperella pyogenes*, and *Mannheimia* sp. were less common. More bacterial species were cultivated from samples from affected ears (13), compared to samples from non-affected ears (9). Farm A had the most diverse bacterial species namely 12. Figure 5 shows a heatmap of all bacteria cultured on agar, with their load, sorted by prevalence.

### Histopathology

**Figure 6:**
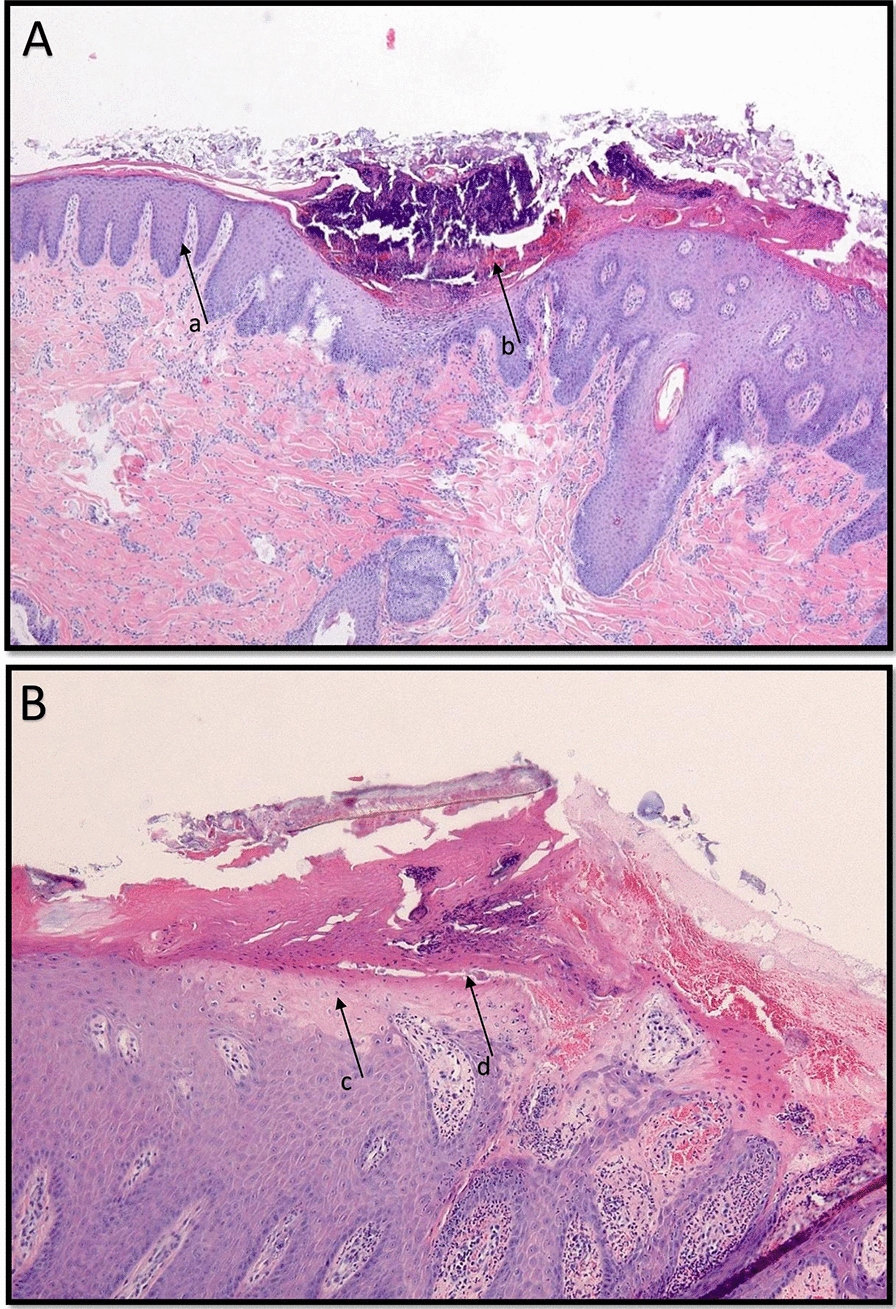
**Microscopic picture of biopsy of affected ear**. **A** Sample with gross lesion score 3. Severe epidermal hyperplasia (a) with focal erosion covered by a serocellular crust (b). **B** Sample with gross lesion score 1. The epidermal pallor of the superficial stratum spinosum (c) and separation of the hyperkeratotic layer (d).

In total, 89 biopsy samples (30 pigs in each farm; one sample from farm C was lost) from ears were analyzed histologically. Epidermal hyperplasia and hyperkeratosis were identified in all afflicted tissue samples. As illustrated in Figure 6, the epidermis was eroded and covered with a crust composed of degenerated leukocytes, nuclear debris, hemorrhage, and coagulated serum proteins (serocellular crust).

In tissues with gross lesion scores 2 and 3, epidermal pallor (hydropic degeneration keratinocytes and/or spongiosis) was often marked adjacent to the hyperkeratosis and was associated with the separation of the hyperkeratotic layer (Figure 6). Neutrophilic epidermal (pustular) and dermal inflammation as well as the formation of granulation tissue was most severe in gross lesion scores 2 and 3. Bacterial coccoid microcolonies were found in numerous samples of afflicted tissue. In three samples (PEN lesion score 2 and 3) and one sample (PEN lesion score 2), vasculitis and thrombosis were present. The histopathological findings of all samples are summarized in Table 2.

**Table 2:** **Prevalence of histopathological lesions in ear biopsies from nursery pigs with different severity of lesions**.

Farm	A (*n* = 30)				B (*n* = 30)				C (*n* = 29)			
Lesion score	0 (*n* = 8)	1 (*n* = 11)	2 (*n* = 11)	3 (*n* = 0)	0 (*n* = 10)	1 (*n* = 12)	2 (*n* = 8)	3 (*n* = 0)	0 (*n* = 10)	1 (*n* = 6)	2 (*n* = 6)	3 (*n* = 7)
Epidermal hyperplasia	2	11	11	–	5	12	8	–	1	6	6	7
Epidermal hyperkeratosis	2	11	11	–	2	12	8	–	5	6	6	7
Epidermal pallor	0	0	1	–	0	5	2	–	0	1	5	2
Epidermal separation	0	8	5	–	0	8	4	–	0	4	6	7
Epidermal erosion	0	11	10	–	0	11	8	–	0	4	6	7
Serocellular crust	1	11	10	–	0	9	7	–	0	5	4	5
Bacterial microcolonies	0	8	5	–	0	4	3	–	0	4	3	6
Inflammation neutrophils	1	11	11	–	0	11	8	–	1	4	6	7
Dermal granulation tissue	0	8	11	–	0	7	8	–	0	3	4	4
Vasculitis	0	0	0	–	0	0	1	–	0	0	1	1
Thrombosis	0	0	0	–	0	0	2	–	0	0	1	1

Modest epidermal hyperplasia and hyperkeratosis were identified in a few samples of animals with no visible ear abnormalities.

### Mycotoxins analysis

At least one mycotoxin was present in plasma samples of 89% of affected and 100% of non-affected piglets. The most frequently found mycotoxins were OTA, ENNB1, and DON (Table 3).

**Table 3:** **Percentage of mycotoxin positive plasma samples (x), and their average concentration (y) [ng/mL] obtained from pigs with and without porcine ear necrosis (PEN)**.

Mycotoxins	Farm	A		B		C	
	PEN	PEN+ (*n* = 21)	PEN− (*n* = 8^a^)	PEN+ (*n* = 20)	PEN− (*n* = 10)	PEN+ (*n* = 19^a^)	PEN− (*n* = 10)
DON	x	71	50	0	0	0	0
	y	0.68	0.64				
OTA	x	52	50	60	80	84	90
	y	0.22	0.20	0.32	0.35	0.48	0.62
ENNB1	x	24	38	0	0	0	0
	y	0.14	0.17				
ENNB	x	90	100	70	100	16	30
	y	0.28	0.37	0.26	0.38	0.17	0.12
TEA	x	38	50	0	0	0	0
	y	1.57	1.88				
AME	x	5	0	5	0	0	0
	y						

DON: deoxynivalenol, OTA: ochratoxin a, ENNB1: enniatin B1, ENNB: enniatin B, TEA: tenuazonic acid, AME: alternariol monomethyl ether.

^a^One sample analysis failed, and one sample got lost.

On farm A, the most prevalent mycotoxins were DON, OTA, and ENNB, followed by ENNB1 and TEA. On farm B, OTA and ENNB mycotoxins were found almost exclusively, and on farm C, the main mycotoxin was OTA. In six out of nine cases, the average concentration of the mycotoxins was lower in PEN-affected animals compared to non-affected pigs.

We failed to reject the null hypothesis for all tested mycotoxins, namely DON (*p* = 0.227), OTA (*p* = 0.266), ENNB1 (*p* = 0.654), ENNB (*p* = 0.084), TEA (*p* = 0.243) and AME (*p* = 0.331). Also, an increase in the number of mycotoxins (isolated from plasma samples) was not associated (Wald χ^2^ = 1.91, *p* = 0.167) with a change in the odds of having an increase in the severity of PEN, with an odds ratio of 0.761 (95% CI 0.517 to 1.121).

Analysis of the feed revealed the presence of four mycotoxins namely DON, FB1 and FB2, and ZEN (Table 4). The feed on farm A was the most contaminated in terms of concentration and number of different mycotoxins.

**Table 4:** **Mycotoxin concentration of feed samples from the nursery unit in each of the three farms (A, B and C)**.

Sample	Farm A		Farm B		Farm C	
	1	2	1	2	1	2
Mycotoxin (µg/kg)						
DON	97.1	132.0	21.8	24.6	27.4	32.3
FUM B1 + B2	70.9	35.5			32.4	51.4
ZEA	20.4	23.4				

One feed sample (1) was taken 1 week post-weaning, the other feed sample (2) was taken 4 weeks post-weaning.

EU reference value in feed for pigs and piglets for deoxynivalenol (DON) is 900 µg/kg, fumonisin (FUM B1 + B2) 5000 µg/kg, and for zearalenone (ZEA) 100 µg/kg, respectively.

### Drinking water analysis and stable climate

The results of the drinking water analyses showed a high concentration of ammonia in farm A, while the water was contaminated with *enterococci* on farms B and C (Table 5). The pH of the drinking water was 3.7, 7.5, and 7.3 in farms A, B, and C, respectively.

**Table 5:** **Results of bacteriological, biochemical, and macroscopic analysis of the drinking water in the three farms**.

Parameter	Farm A	Farm B	Farm C	Laboratory reference values
Macroscopic evaluation				
Physical appearance	Bright	Bright	Brown sediment	Bright
Smell	Light smell	No	Light smell	No
Color	Colorless	Colorless	Colorless	Colorless
Bacteriological analysis				
Number of coliforms (cfu/mL)	0	0	10	< 100
Intestinal enterococci (cfu/100 mL)	2	> 100	> 100	< 1
Sulfite-reducing clostridia (cfu/20 mL)	0	0	21	< 1
Aerobic bacteria (22 °C) in total (cfu/mL)	1010	1300	7000	< 100 000
Aerobic bacteria (37 °C) in total (cfu/mL)	650	13	800	< 100 000
Chemical analysis				
pH	3.7	7.5	7.3	4–9
Ammonia (mg/L)	145.7	0.2	0.9	≤ 2
Nitrates (mg/L)	69.0	< 10.0	12.0	≤ 200
Nitrites (mg/L)	< 0.1	< 0.1	< 0.1	≤ 0.5
Sulfates (mg/L)	115.0	81.0	20.0	≤ 250
Total hardness (°D)	29.9	13.0	4.4	≤ 20
NaCl (mg/L)	93.0	69.0	27.0	≤ 3000

**Figure 7:**
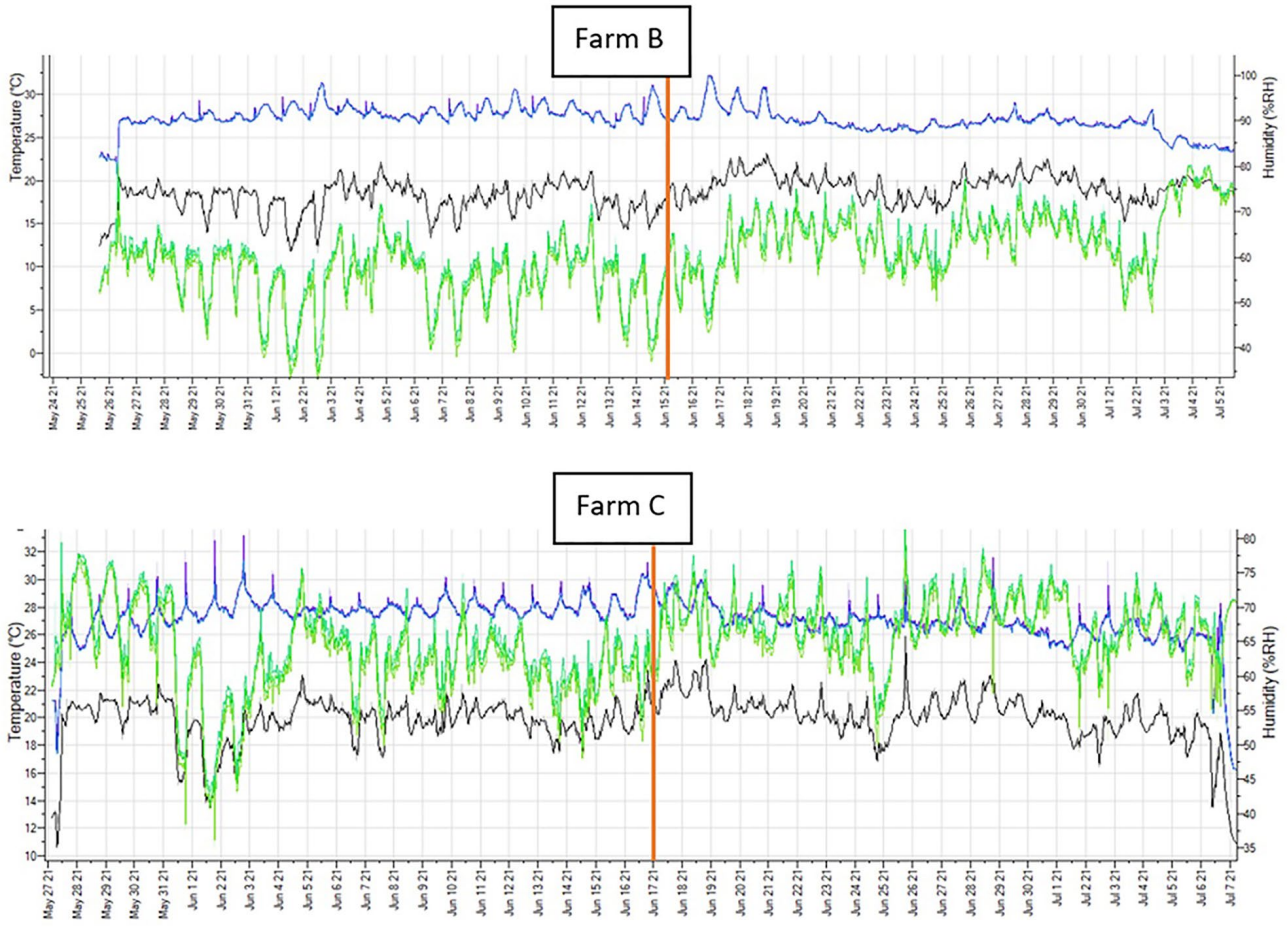
**Two graphs illustrating temperature (blue line), humidity (green line), and dew point (black line) measured in the compartment during the nursery period on farms B and C**. The red vertical line is a timepoint when the first PEN lesions started to appear.

The results of the stable climate measurements on farms B and C were as follows: average temperature (± SD): farm B 27.5 °C (± 1.1), farm C 27.3 °C (± 1.0), relative humidity (± SD): farm B 59% (± 8), farm C 65% (± 6) (Figure 7).

## Discussion

The present study applied a wide variety of diagnostic approaches to assess the pathogens involved in PEN and characterize PEN lesions in nursery pigs from three farms. The ear lesions appeared at 3 to 4 weeks after weaning, but the prevalence and severity varied between farms and pens within a single weaning batch. Many different bacteria were identified by nanopore sequencing as well as by bacterial cultures, including *Staphylococcus*, *Fusobacterium*, and *Mycoplasma*. The main histopathological changes were epidermal splitting of the corneum and spinosum, hyperplasia, hyperkeratosis, and ulceration of the epidermis with serocellular crusts. They were observed primarily in the outer layer of the epidermis, suggesting that damage is initiated from the outer part of the skin.

### Prevalence of lesions

The PEN lesions appeared in pigs of 7 to 9 weeks of age, or 3 to 4 weeks post-weaning. This is similar to previous studies [2, 17]. None of the animals showed PEN lesions at weaning. The highest prevalence of 50% was reached on farm C. This is higher than in our previous study but lower than the 80–100% prevalence reported [18, 19]. The number of affected animals steadily increased towards the end of the nursery on farm A, while there was a slight decrease in the last 2 weeks in the other two farms. Farm C, with the highest floor space availability (0.33 m^2^/piglet—see Additional file 1), had the highest prevalence and severity of ear lesions, the prevalence in the other two farms with less floor space for the pigs was 27% (farm A) and 12% (farm B). There was a large variation in the prevalence of PEN between the pens of a compartment. Pens with a high and low prevalence of PEN lesions were often found next to each other. This was also observed in previous studies [2, 20], and indicates that it is important to include different pens to assess the overall prevalence in a compartment.

Apart from PEN lesions, the study also showed that many pigs were affected by abrasion-like lesions or scratches during the first week after weaning. These lesions healed very fast and were different from the PEN lesions which occurred later. The former lesions are likely due to aggressive biting, targeting mainly the head, and front body part of animals. Such biting behavior may occur during the hierarchy formation after regrouping animals, and/or when competing for feed [21]. The present study also clearly shows that the presence of an ear tag (close to the ear tip/edge) decreased almost 5 times the risk of ear mild lesions/scratches occurrence shortly after weaning. Further research is needed to confirm this result and elucidate whether biting or other mechanisms may be involved in PEN.

### Severity of lesions

The results showed a large variation in PEN lesion severity between pigs and between farms. Farms A and B were affected mainly with lesion score 1 (88–97%), whereas Farm C was most severely affected. Half of the animals had lesions with a score of 2, and one-fifth had lesions with a score of 3. Several affected pigs had lost parts of the ear tissue and suffered from large moist wounds.

This is more severe compared to the previous study on a different farm in which 85% of affected animals developed lesions scored as 1, and 14% with score 2. The study of Pejsak [10] noticed an overall PEN prevalence of 11–13%, with 7–8% for lesions corresponding to score 2 presented in the present study. In terms of the severity, the current study clearly showed the protective effect of an ear tag.

### Results of nanopore metagenomic sequencing in serum and scrapings

The nanopore metagenomic sequencing of serum samples revealed the presence of several pathogens, mainly viruses. The high PPV2 and PPV5 loads found on farms B and C in both PEN-affected and non-affected animals point to an ongoing viremia. There are currently seven (1–7) PPVs described, with PPV1 being the cause of stillbirth, mummification, embryonic death, and infertility [13], but the role of PPV2-7 is not well investigated [22, 23]. Some old studies link the presence of PPV (NADL-8 and Kresse strains) to skin lesions [24, 25], as in this case, there are actively growing cells with a high mitotic index, creating favorable conditions for these viruses. However, only 11% of ear scrapings in the current study (Farm B and C) contained PPV. Torque teno sus virus was another virus found in serum, but also in this case, no association with the severity could be established. TTSuV is found all over the world [26, 27] there is no clear link between the virus and any clinical signs [28].

The nanopore sequencing results of the ear scrapings revealed a wide range of viruses and bacteria in the three farms, present in the lesions as well as on the healthy skin. The predominant viruses were bocaparvovirus, various parvovirus types, and TTSuV. Other viruses such as astrovirus, picobirnavirus, and rotaviruses were abundantly detected, but these are thought to be rather environmental contaminants [13]. Bocaparvovirus was first detected by Blomström et al. [29], but its pathogenicity remains unclear till now [30, 31].

Bacterial genera belonging to *Streptococcus*, *Staphylococcus*, *Fusobacterium*, *Mycoplasma*, and *Clostridium* were identified in many samples. In samples of affected animals, the bacterial loads were higher than in samples from pigs without PEN lesions. Moist and bloody wounds are a good environment for bacterial growth, so it is not clear whether the bacteria have an etiological role or whether the high load is a result of the favorable environment to multiply. Environmental contaminating bacteria such as *Prevotella* sp. and *Bacteroides* sp. were also identified [32].

*Clostridium* and *Fusobacterium* are anaerobes that can infect open wounds and are common in the environment. The relatively high prevalence in the studied samples (53–56%) may be indicative of their potentially important role in PEN. *Fusobacterium necrophorum* is known for its ability to cause skin lesions or necrosis in various animal species [33], whereas *Clostridium perfringens* is able to induce myonecrosis and gas gangrene [34]. At histology, bacteria with a morphology of *Clostridia* sp. (rod-shaped) or *Fusobacterium* sp. (filamentous) invading the dermis were not observed. The *Staphylococcus* and *Streptococcus* species found in many samples might be involved in the pathogenesis. However, microcolonies of cocci were mainly observed associated with a superficial serocellular crust. Likely they might act as secondary invaders of the wounds. *Mycoplasma* sp. (most likely *M*. *hyopharyngis*) was found in 83% of affected ears and was absent in non-affected ears. This bacterium is poorly described in the scientific literature. Kobisch and Friis reported its presence in the pharynx of a pig in 1996 [35], but no information about its possible pathogenicity has been released since then. The presence of this bacterium on affected ears might originate from ear-biting, or at least contact between the mouth of pigs and the ears of pen mates. Moreover, this suggests ear-biting might be involved in or contribute to the development of PEN lesions. Park et al. [6] discussed ear-biting as a potential cause of PEN. These findings, combined with ear-biting and chewing observed during the farm visits in the present study, may indicate the importance of the behavioral component in the genesis of severe PEN lesions, although further research is needed to confirm this. Weissenbacher-Lang [19] investigated the presence of *M. suis* by PCR in 9 PEN-positive farms, but only 2 out of 72 samples were positive. In the present study, *Mycoplasma suis* was not detected, nor were the associated clinical signs (hemolytic anemia and icterus with fever) or the lesions (vascular thrombosis and coagulopathy) [36, 37]. Therefore, the importance of *M. suis* in the pathogenesis of PEN is rather limited in the current study.

### Bacteriology

Bacteriological culturing on agar revealed that more diverse bacteria were detected in samples from affected than in non-affected ears. The most common bacteria were *Streptococcus dysgalactiae* ssp. *equisimilis* (77%), *S. suis* (30%), *Staphylococcus aureus* (73%), and *S. hyicus* (33%).

*Streptococcus dysgalactiae* ssp. *equisimilis* belongs to β-hemolytic streptococci and has been related to endocarditis, arthritis, and meningitis. The main route of infection is vaginal secretion where the transmission occurs mainly via injuries to the feet and skin lesions [38]. *S. suis* colonizes pigs during parturition. Clinical signs include arthritis, meningitis, and septicemia. Most pigs carry the pathogen without showing any clinical signs [39]. *S. aureus* and *S. hyicus* can be commonly found on the skin surface of healthy pigs [40]. Both pathogens can produce exfoliative toxins that can damage the epidermis. However, in many lesions, epidermal pallor (hydropic degeneration keratinocytes and/or spongiosis) with subsequent separation of the epidermis was present, which differs from the splitting of the epidermis at the level of the stratum granulosum mediated by exfoliative toxins.

A difference has been also found by Weissenbacher-Lang [19] in bacterial loads of streptococci and staphylococci between affected and non-affected animals. Those cocci can have a negative effect on the injured epidermis. Costa et al. [11] aimed to reproduce PEN lesions experimentally by using material obtained from affected animals. The inoculum contained a high concentration of *Staphylococcus* spp. and *Streptococcus* spp. However, only mild lesions and inflammation were induced, which disappeared within 21 days after inoculation.

Metagenomic sequencing has been shown to allow accurate and semi-quantitative detection of bacteria in different types of samples [13, 15]. For most of the bacteria, there was an overall moderate to good correlation with the results of the bacterial culture. However, there were also some differences. Metagenomic sequencing allowed us to show the presence of difficult-to-grow bacteria such as *Mycoplasma*, *Fusobacterium*, and *Campylobacter* species. Differences with bacteriological culture were also observed for *Streptococcus* and *Staphylococcus*, which might be related to the growth characteristics of these bacteria. It could be because of their difficult-to-lyse Gram-positive and easy-to-(over)grow features, respectively. Finally, the sampling procedure for both diagnostic assays was different, using scrapings for nanopore metagenomics and swabs for bacterial cultures. Hence this might also impact the final output.

### Gross lesions and histology

The gross lesions were similar on the three farms. The principal histological findings included epidermal splitting of the corneum and spinosum as well as hyperplasia, hyperkeratosis, and ulceration of the epidermis with serocellular crusts together with the presence of bacteria microcolonies and neutrophils and neutrophils in the dermis. Vasculitis, thrombosis, and the involvement of cartilage occurred in less than 5% of affected tissue samples. These findings are not suggestive of an acute trauma affecting the ear auricle, but merely the influence of more repetitive external damage in combination with bacterial infection and toxin production.

The mild lesions differ slightly from those defined by others [1, 41]. They found intra-epidermal abscesses, intracellular keratinocyte edema, para-keratotic hyperkeratosis of the stratum corneum, neutrophil infiltration, vacuolar degeneration and necrosis of basal cells, and the subsequent formation of intra-epidermal vesicles. A focal epidermal necrosis in the early stages, with subsequent extension to broader areas, degeneration of the blood vessels, and vesicular dermatitis was reported. Vesicular dermatitis was not found in the present study [19].

### Mycotoxins

The mycotoxins OTA and ENNB were found in plasma samples on all farms. Other detected mycotoxins in plasma such as DON, ENNB1, and TEA were only found in farm A. The feed analysis data also showed that farm A had the highest level of mycotoxins contamination (DON, FUM B1 + B2, and ZEA). On farm A, the DON concentrations in the feed samples were 97 and 130 µg/kg, and DON was detected in 65% of the plasma samples. In a previous study [2], DON concentrations in the feed were 60 to 175 g/kg, whereas it was not detected in plasma.

There were no major differences in the mycotoxin levels in the plasma of PEN-affected and non-affected animals. This corroborates with previous studies [2, 19]. Actually, in six out of nine samples, the average concentration was higher in the animals without PEN lesions. No associations between mycotoxins in the plasma and PEN severity score were seen.

### Drinking water, and stable climate

The results of the drinking water analyses showed a high concentration of ammonia in farm A and contamination with *enterococci* on farms B and C. The origin of the high ammonia concentration is not clear, but it might be related to the acidification procedure or the use of fertilizers in the close area to the farm, as deep drainage water was used. Contamination with sewage or animal wastes is less likely in this case, as much higher bacterial contamination would be expected [42]. The high levels of enterococci can point to contamination of the drinking water with manure [43]. The drinking water quality should be improved, but the role of deviations in these parameters in the PEN problems in these farms is not clear. Deviations are also often seen in other farms without PEN problems.

No major abnormalities were present in ambient temperature and relative humidity in the nursery units of farms B and C. The relative humidity slightly increased in the week when PEN lesions appeared. The increase was minor and likely not relevant to the present study.

The evolution of PEN lesions was similar in the three farms. The lesions appeared 3 to 4 weeks after weaning and peaked towards the end of the nursery. The prevalence and severity of the lesions varied substantially between pens. The most abundant bacteria detected by nanopore sequencing were *Staphylococci*, *Streptococci*, *Clostridium*, *Fusobacterium*, and *Mycoplasma.* Interestingly, bacteria, namely *Mycoplasma hyopharyngis*, was only detected in PEN-affected pigs. Histopathological lesions were mainly found in the outer layer of the epidermis. Therefore, the results suggest that the suckling, chewing, or biting of pigs on the ear of pen mates followed by bacterial infection and skin damage may be important in the pathogenesis. Further research focusing on monitoring the behavior of pigs throughout the nursery phase is therefore warranted.

### Supplementary Information

**Additional file 1. Questionnaire results**. Characteristics of the farms.

**Additional file 2. Metagenomic analysis results.** Full results of the metagenomic analysis performed on ear scrapings of affected and non-affected pigs.
